# Comparative Investigation of Methods for Analysis of SARS-CoV-2-Spike-Specific Antisera

**DOI:** 10.3390/v14020410

**Published:** 2022-02-17

**Authors:** Marie-Luise Herrlein, Sascha Hein, Tobias Zahn, Ines Mhedhbi, Jan Raupach, Younes Husria, Nuka Ivalu Benz, Jonathan Eisert, Daniela Bender, Vanessa Haberger, Florian D. Hastert, Lisa Henss, Barbara S. Schnierle, Julia C. Stingl, Michael Dreher, Eberhard Hildt

**Affiliations:** 1 Paul-Ehrlich-Institut, Department of Virology, Paul-Ehrlich Street 51-59, D-63225 Langen, Germany; marie-luise.herrlein@pei.de (M.-L.H.); sascha.hein@pei.de (S.H.); tobias.zahn@pei.de (T.Z.); ines.mhedhbi@pei.de (I.M.); jan.raupach@pei.de (J.R.); younes.husria@pei.de (Y.H.); nuka.benz@pei.de (N.I.B.); jonathan.eisert@pei.de (J.E.); daniela.bender@pei.de (D.B.); vanessa.haberger@pei.de (V.H.); florian.hastert@pei.de (F.D.H.); lisa.henss@pei.de (L.H.); barbara.schnierle@pei.de (B.S.S.); 2 Institute of Clinical Pharmacology, RWTH Aachen University Hospital Aachen, Pauwelsstraße 30, D-52074 Aachen, Germany; jstingl@ukaachen.de; 3 Department of Pneumology and Intensive Care Medicine, RWTH Aachen University Hospital Aachen, D-52074 Aachen, Germany; mdreher@ukaachen.de

**Keywords:** COVID-19, SARS-CoV-2, convalescent sera, ARDS, neutralization assay, humoral immune response

## Abstract

In light of an increasing number of vaccinated and convalescent individuals, there is a major need for the development of robust methods for the quantification of neutralizing antibodies; although, a defined correlate of protection is still missing. Sera from hospitalized COVID-19 patients suffering or not suffering from acute respiratory distress syndrome (ARDS) were comparatively analyzed by plaque reduction neutralization test (PRNT) and pseudotype-based neutralization assays to quantify their neutralizing capacity. The two neutralization assays showed comparable data. In case of the non-ARDS sera, there was a distinct correlation between the data from the neutralization assays on the one hand, and enzyme-linked immune sorbent assay (ELISA), as well as biophysical analyses, on the other hand. As such, surface plasmon resonance (SPR)-based assays for quantification of binding antibodies or analysis of the stability of the antigen–antibody interaction and inhibition of syncytium formation, determined by cell fusion assays, were performed. In the case of ARDS sera, which are characterized by a significantly higher fraction of RBD-binding IgA antibodies, there is a clear correlation between the neutralization assays and the ELISA data. In contrast to this, a less clear correlation between the biophysical analyses on the one hand and ELISAs and neutralization assays on the other hand was observed, which might be explained by the heterogeneity of the antibodies. To conclude, for less complex immune sera—as in cases of non-ARDS sera—combinations of titer quantification by ELISA with inhibition of syncytium formation, SPR-based analysis of antibody binding, determination of the stability of the antigen–antibody complex, and competition of the RBD-ACE2 binding represent alternatives to the classic PRNT for analysis of the neutralizing potential of SARS-CoV-2-specific sera, without the requirement for a BSL3 facility.

## 1. Introduction

After its emergence in December 2019, severe acute respiratory syndrome coronavirus 2 (SARS-CoV-2) rapidly spread all over the world. SARS-CoV-2 is an enveloped virus. The positive-sense, single-stranded RNA genome has a size of approximately 30 kb. In the family *Coronaviridae*, SARS-CoV-2 belongs to the genus *Betacoronavirus*. Up to now, SARS-CoV-2 has caused more than 259 million cases of coronavirus disease 2019 (COVID-19) (source: COVID-19 Dashboard by the Center for Systems Science and Engineering (CSSE) at Johns Hopkins University (JHU)). Although many infections, especially those of younger people, are asymptomatic or characterized by mild symptoms, respiratory distress in the more severe cases frequently requires intensive care treatment and mechanical ventilation. At present, there are more than 5.1 million deaths relating to SARS-CoV-2 infections worldwide (source: COVID-19 Dashboard by the Center for Systems Science and Engineering (CSSE) at Johns Hopkins University (JHU)). Currently, there are several vaccines approved by the regulatory authorities based on emergency use authorization (EUA) or conditional approval. The vaccines (mRNA or adenoviral vectors) encode the spike protein (S) of SARS-CoV-2 as an antigen. S is a glycoprotein which forms trimers and is located on the viral surface [1]. During the entry process, S mediates the interaction with the receptor-angiotensin-converting enzyme 2 (ACE2) [2,3]. The S-monomer encompasses two domains, S1 and S2. The S1 domain contains the receptor-binding domain (RBD), which is a major target for neutralizing antibodies [4]. The S protein is the target of almost all neutralizing antibodies found in the convalescent sera or elicited by vaccines. In addition to the RBD-targeting antibodies, there are neutralizing antibodies, which bind to an antigenic “supersite” in the N-terminal domain involving residues 141 to 156 and residues 246 to 260 [5,6].

Mutations found in variants of concern (VOC) are frequently located in the S protein. This could represent a selection for evasion of antibody responses and might impact the capacity of preexisting antibodies to neutralize the virus [7].

In contrast to this, there is evidence that the recognition of T-cell epitopes is almost not affected by these mutations [8].

In addition to S, the viral N protein induces high levels of antibodies during infection. The N protein is involved in the packaging of the viral RNA and in its transcription. Many serological assays for detection of SARS-CoV-2-specific antibodies are based on the N protein. Although there are high N-specific titers, the neutralizing capacity of these antibodies might be limited, as N is not directly involved in the entry process and it is shielded by the envelope proteins [9].

Infection- and vaccine-elicited immune responses are multifaceted, but in many cases, there might be a correlation between antibody responses and the level of protection. In light of this, there is an urgent need to study and, if possible, to establish an antibody-based correlate of protection [10]. This would allow a better characterization and comparison of vaccines and would be of prognostic value for convalescent and vaccinated individuals to decide whether there is still a protective immune response. Moreover, a defined correlate of protection would be a central factor for the assessment of immunogenicity data of vaccine candidates for marketing authorization.

This would require robust methods to quantify the titer of neutralizing antibodies. The gold standard for determination of neutralizing antibodies is a plaque reduction neutralization assay (PRNT) [11]. In the case of SARS-CoV-2, this assay requires access to a BSL3 facility and is a labor-intensive process. Pseudotyped lentiviral vectors are viral particles that contain the spike protein on their surface and harbor a reporter gene, which allows the quantification of the neutralization activity. The comparability of pseudotype-based and PRNT assays has been shown in previous studies [12,13]. ELISAs represent a convenient and rapid method to quantify and qualify antibodies with respect to their class. However, there is not necessarily a stringent correlation between binding and neutralizing antibodies [14]. Surface plasmon resonance (SPR) methods can be used for quantification of antibodies and to characterize the binding in more detail, i.e., by determining the stability of the antigen–antibody complexes or the affinity. These parameters are of major relevance with respect to the neutralizing capacity of antibodies/antisera. Moreover, SPR-based approaches allow in detail analysis of the potential of sera to compete the S/ACE2 interaction—a relevant aspect for neutralizing antibodies. A limitation might be that purified proteins or fragments thereof are used. This setting could affect the conformation of spike and/or ACE2. In case of the syncytium formation assay (SFA), ligand (S) and receptor (ACE2) are both embedded in a membrane environment that facilitates proper folding. If there is an interaction between S (on the surface of spike-positive cells) and ACE2 (on the surface of ACE2-positive cells), the spike-immanent fusogenic activity causes fusion of the ACE2-expressing cells and of the spike-expressing cells, which can be easily quantified. RBD-binding antibodies can compete this interaction [15,16].

This study aims to compare various methods for quantification of the antibody response. For this purpose, we analyzed sera from hospitalized patients suffering from COVID-19, with or without ARDS by ELISA, neutralization assays, SPR and SFA.

## 2. Materials and Methods

### 2.1. Cell Culture

HEK293T cells (ATCC CRL-3216™) and Vero E6 (ATCC^®^ CRL-1586™) cells were maintained in Dulbecco’s modified Eagle’s medium (DMEM, Sigma, Taufkirchen, Germany), supplemented with 10% FCS (Sigma, Taufkirchen, Germany), 1% penicillin/streptomycin and 1% L-glutamine and incubated with 5% CO_2_ at 37 °C. Cells were infected using the SARS-CoV-2 UVE/SARS-CoV-2/2020/FR/702 isolate (here named as MRS). The identity and integrity of the furin cleavage site was analyzed by PCR according to the protocol of Lau et al. [17]. The isolate was provided by the European Virus Archive GLOBAL (EVAg).

### 2.2. Protein Production and Purification

For production of RBD in HEK293T cells, a pcDNA3.1-based expression vector was generated. For this purpose, the *rbd*-encoding sequence was amplified from the pCAGGS-sRBD plasmid (Florian Krammer, Icahn School of Medicine at Mount Sinai [18]) and inserted into the expression pcDNA3.1(-)-plasmid (Thermo Fisher Scientific, Waltham, MA, USA) using standard cloning procedures. The plasmid encodes for a fusion protein of the wildtype spike RBD, with a C-terminal hexa histag. DMEM medium, supplemented with 10% FCS, was used for cultivation of HEK293T cells. Cells were seeded at 4.5 × 10^6^ cells/150 mm dish and transfected 6 h later by mixing 15 µg plasmid DNA (pcDNA3.1(-)-SCov2-RBD) with 1.5 mL of polyethylenimine (PEI) in 1.5 mL PBS (60 µg/mL). A duration of 16 h after transfection, cells were supplied with 30 mL fresh medium. The supernatant was harvested 72 h post transfection. For purification, a 5 mL Ni-NTA affinity chromatography column (Cytiva, Washington, DC, USA) was equilibrated with 5 column volumes (CV) of wash puffer with a flow rate of 2 mL/min. The sterile-filtered supernatant was mixed 1:1 with wash buffer (40 mM imidazole in PBS, pH 8.0) before loading on the column. The column was washed with 15 CV of wash buffer. The elution of the protein was performed with 250 mM imidazole in PBS. For buffer exchange with PBS, centrifugation at 4 °C and 4000× *g* using a 10 kDA Amicon Centrifugal Filter (Merck Millipore, Burlington, MA, USA) was performed.

### 2.3. Plaque Reduction Neutralization Test (PRNT_50_)

For the PRNT (plaque reduction neutralization test) Vero Er cells were used; 6 × 10^5^ of Vero E6 cells per well of a 6-well plate (Greiner Bio-One, Kremsmünster, Austria) were seeded and incubated for 24 h at 37 °C. For the analysis, the sera were 2-fold serially diluted (1:20 to 1:640) and incubated with 80 PFU of the early isolate (MRS) in a total volume of 100 µL at 37 °C for 1 h. In the next step, the medium was removed and replaced by 1 mL of fresh DMEM without FCS. The complete virus–serum mix was added to the cells and incubated at 37 °C for 1 h. During incubation, the 6-well plate was agitated every 15 min. After incubation, the supernatant was removed and 2 mL of 0.4% SeaPlaque Agarose (Biozym, Hessisch-Oldendorf, Germany) in DMEM containing 5% FCS, 1% penicillin/streptomycin and 1% L-glutamine was given to the infected cells. After 2 days, the cells were fixed for 20 min with a 4% formaldehyde solution in PBS and stained with 1 mL 0.1% crystal violet in 20% ethanol for 15 min. Finally, after removal of the staining solution, the wells were washed with 2 mL distilled water and the plaques were counted. The PRNT titer was calculated based on a 50% reduction in plaque counts (PRNT_50_). The PRNT_50_ titer was chosen according to a WHO guideline [19]. As compared with higher cutoffs (PRNT_90_), the determination of the PRNT_50_ enables more accurate results as the titers are based on the linear part of the titration curve.

### 2.4. Pseudotype-Based Neutralization Assay

For generation of lentiviral vectors HEK293T cells were co-transfected with HIV-1 gag/pol, rev, the luciferase-encoding lentiviral vector genome and the SARS-CoV-2 delta 19 spike (#MN908947), using Lipofectamine^®^ 2000 (Thermo Fisher, Darmstadt, Germany), as described previously [20]. The SARS-CoV-2 gene was commercially synthesized (Eurofins, Ebersberg, Germany) and cloned into a pIRES-GFP vector, as described before [21]. After harvest and concentration by ultracentrifugation, vector particles were stored at −80 °C. Pseudotyped vectors and serially diluted human serum (1:60 to 1:14,580) were incubated in triplicates for 45 min at 37 °C and then used to transduce HEK293T-hACE2 cells [22]. After 48 h, Britelite plus luciferase substrate (PerkinElmer, Waltham, MA, USA) was added to measure luciferase activity. For each sample, the reciprocal area under the curve (AUC) value was calculated. The AUC values were calibrated to the WHO international standard and international units/mL are indicated as neutralization activity.

### 2.5. Sera

Convalescent sera (Table 1) from hospitalized patients suffering from ARDS or not were provided by the RWTH Centralized Biomaterial Bank (RWTH cBMB) of the Medical Faculty of RWTH Aachen University. The two sera (N11801 and N11802) from the PEI reference panel, which were collected 2017, and sera from March 2020 to June 2020 from SARS-CoV-negative persons were used as negative control. These sera were considered as negative in the described assays. The cutoff for the PRNT titer is based on a 50% or greater reduction in plaque counts (PRNT_50_).

### 2.6. Ethics

The convalescent sera were provided by the RWTH cBMB (RWTH Aachen University). The provisions of the Declaration of Helsinki and good clinical practice guidelines were observed. The standing orders of the cBMB and the approval by the local ethics committee (Ethikvotum 206/09 der Ethikkommission der Medizinischen Fakultät der RWTH Aachen University) were considered when performing the studies.

### 2.7. ELISA

A measure of 50 µL purified antigen (concentration of 4 µg/mL RBD in PBS) was used to coat the 96-well microtiter plates (costar 3590, Corning Incorporated, Kennebunk, ME, USA) over night at 4 °C. Afterwards, the plates were blocked for 1 h at RT with 10% FCS in PBS. Between each step, the plates were washed 3 times with PBS-T (0.05% Tween). The sera were pre-diluted 1:100 in 10% FCS in PBS and incubated for 1.5 h at RT. For detection, anti-human IgG-, IgM- or IgA-HRP-linked ECL Ab (Cytiva, Dassel, Germany) was used in a 1:3000 dilution. Between each step, the plates were washed 3 times with PBS-T (0.05% Tween). The ELISA plates were developed with 75 µL TMB ELISA Substrate Solution (eBioscience, San Diego, CA, USA) for 5 min, stopped with 75 µL 1 N sulfuric acid and analyzed directly at 450 nm on a Tecan reader (Tecan Group, Männedorf, Schweiz).

### 2.8. Surface Plasmon Resonance

Surface plasmon resonance using the Biacore T200 system (Cytiva, Marlborough, MA, USA) was applied for analysis of the sera. After 1:10 dilution (20 µM final concentration) of the purified RBD domain in immobilization buffer (10 mM HEPES + 0.05% Tween-20, pH 7.0), it was immobilized on a CM5 sensor chip (Cytiva, Marlborough, MA, USA). The final response level for the RBD was 6039.9 RU, while on the same chip, 1 flow-cell remained blank. This was done to enable background subtraction. For 1:20 dilution of the sera running buffer (10 mM HEPES pH 7.1, 3 mM EDTA, 150 mM NaCl + 0.05% Tween) supplied with 10% NSB reducer (Cytiva, Marlborough, MA, USA) was used. Unspecific binding was blocked by running three start-up cycles with negative serum, before patient sera were applied. Contact time was set to 180 s, followed by 180 s dissociation. Subsequently, ACE2 (Sino Biological, Beijing, China) was added at a concentration of 0.1 µM for 60 s to analyze the competition of the sera with RBD-ACE2 binding, as described by Ju et al. [23]. Two regeneration steps were performed using 10 mM Gly-HCl pH 2.5 for 60 s and 30 s, respectively. For the control values, anti-RBD antibodies (Sino Biological, Peking, China; or produced in our lab) as well as anti-His (Santa Cruz Biotechnology, Dallas, TX, USA) were used at the beginning and end of each run as well as five times distributed evenly throughout the run.

Using the Biacore T200 evaluation software (Cytiva, Marlborough, MA, USA), adjustment of patient serum responses for the anti-RBD controls was performed to compensate for decrease in binding capacity of the surface over time. Anti-His controls were used for adjustment of ACE2-binding responses. This antibody was used, as the ACE2 binding is not affected by the anti-His antibody.

### 2.9. Cell–Cell Fusion Assay

To analyze the neutralizing capacity of the convalescent sera on cell–cell fusion and syncytia formation, HEK293T cells transfected with the SARS-CoV-2 spike protein and Vero cells were co-cultured in an assay adapted from Theuerkauf et al. [16]. Briefly, HEK-cells were trypsinized 36 h after transfection, adjusted to 5 × 10^4^ cells in 50 µL Dulbecco’s Modified Eagle’s Medium (DMEM, Sigma-Aldrich, St. Louis, MO, USA), and mixed with 50 µL of 1:1 diluted serum. After 30 min incubation at 37 °C, the same amount of Vero cells was added and the mixture was seeded into 8-well chamber slides (Ibidi, Gräfelfing, Germany). Cells were cultured another 24 h, before they were fixed with 4% formaldehyde (Carl Roth, Karlsruhe, Germany) in PBS.

For subsequent immunofluorescent staining, cells were permeabilized for 10 min with 0.5% Triton X-100 (Sigma-Aldrich, St. Louis, MO, USA) in PBS and blocked for 15 min with 1% BSA (Carl Roth, Karlsruhe, Germany) in PBS. Following 1 h incubation with an anti-RBD antibody (1:500, Sinobiological, Peking, China), anti-rabbit-Cy3 (Jackson Immuno Research) was applied as secondary antibody for 1 h at room temperature. 4′,6-Diamidino-2-phenylindole (DAPI) (Carl Roth, Karlsruhe, Germany) was used for staining of nuclei, the actin cytoskeleton was visualized with Phalloidin-Atto 633 (Sigma-Aldrich, St. Louis, MO, USA). The stains were analyzed using the confocal laser scanning microscope SP8 (Leica, Wetzlar, Germany). For each condition, the ratio of nuclei that are part of SARS-CoV-2-spike-positive syncytia to the total amount of nuclei was quantified in three fields of view.

### 2.10. Statistical Analysis

For statistical analysis, the software Prism 8 (GraphPad) was instrumental using the default setting. In terms of the SFA, the significance of the results was determined by two-way ANOVA.

### 2.11. Overview

**Figure 1:**
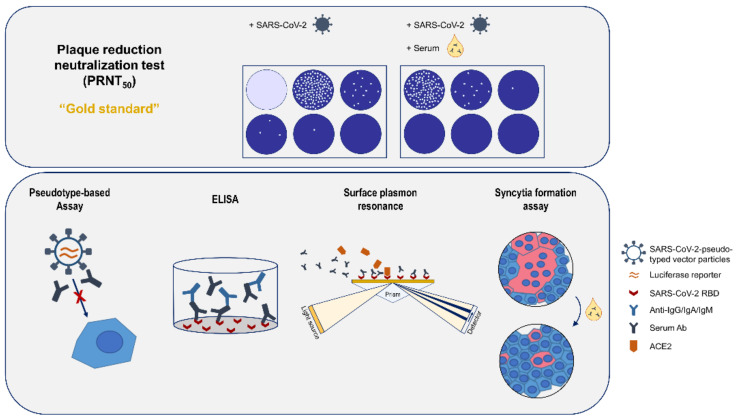
Schematic design of the study.

A schematic overview of all analytic methods used in this study is given in Figure 1.

## 3. Results

The analysis of SARS-CoV-2-spike-protein-specific antibodies is relevant for investigation of the humoral immune response to SARS-CoV-2 due to vaccination or natural infection. The aim of this study was to compare various methods for quantification of the antibody response. For this purpose, we analyzed sera from hospitalized patients suffering from COVID-19 with or without ARDS by ELISAs, neutralization assays, surface plasmon resonance spectroscopy and syncytium formation assay (SFA).

### 3.1. Quantification of Neutralizating Antibodies by Plaque Reduction Neutralization Test

Neutralization assays represent the gold standard for characterization of sera with respect to their neutralizing capacity [11]. Sera from hospitalized patients suffering from ARDS and sera from non-ARDS patients were characterized by plaque reduction neutralization test (PRNT) and by pseudotyped lentiviral vector neutralization assays.

**Figure 2:**
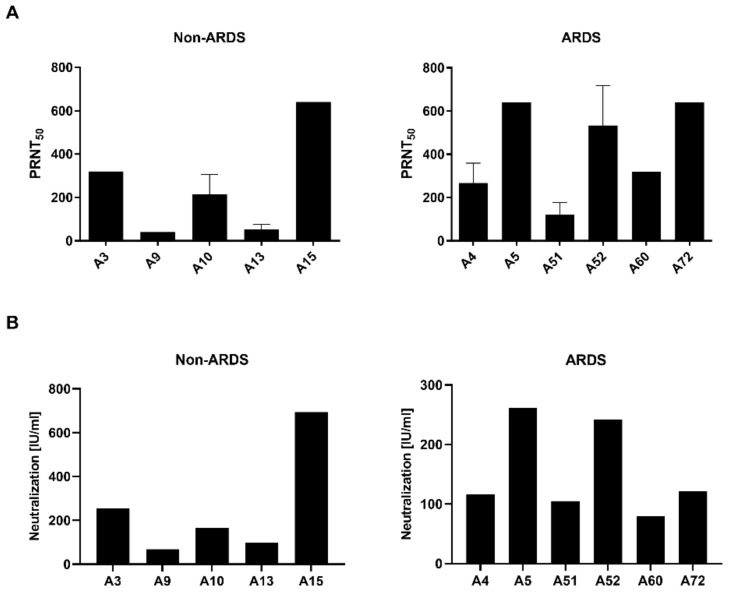
Neutralization assays. (**A**) Plaque reduction neutralization test (PRNT_50_). Titers of convalescent sera from five non-ARDS (left) and six ARDS patients (right) were determined. Two-fold serial dilutions of the sera were incubated with 80 PFU of SARS-CoV-2 MRS, before they were added to Vero E6 cells and a plaque assay was performed. Neutralization is represented by the PRNT_50_ (the 50% plaque reduction neutralization titer, the reciprocal of the 50% inhibitory dilution per serum). (**B**) Pseudotyped lentiviral vector-based neutralization assay. Serial dilutions of convalescent sera were incubated with SARS-CoV-2-pseudotyped vector particles and neutralizing titers were determined by detection of relative luciferase activity and calibrated to international units.

The PRNT (Figure 2A) revealed for the sera derived from the non-ARDS patients one serum (A15) with a high neutralization titer (PRNT_50_ = 640), two sera (A3 and A10) with a moderate neutralizing capacity (A3: 320; A10: 213) and two sera (A9 and A13) with a low neutralizing capacity (A9: 40; A13: 53). Qualitative comparable results were obtained by the pseudotyped lentiviral vector neutralization assay (Figure 2B). For the non-ARDS serum A15, a high neutralization capacity was found (693.67 IU/mL), for the sera A3 (254.58 IU/mL) and A10 (166.48 IU/mL), a moderate neutralizing effect was found, and for the sera A9 (68.09 IU/mL) and A13 (99.10 IU/mL), a low titer was found.

For the ARDS sera, a comparable pattern was found in the PRNT and pseudotype-based assay. In the PRNT assay, sera A5 (PRNT_50_ = 640), A52 (533) and A72 (640) showed a strong neutralizing effect, the sera A4 (267) and A60 (320) showed a moderate neutralizing effect, and sera A51 (120) showed a low neutralization capacity. As for the non-ARDS sera in the pseudotype-based assay (Figure 2B), qualitatively comparable results were obtained. For the sera A5 (261.10 IU/mL) and A52 (242.15 IU/mL), high titers were identified. The sera A4 (116.65 IU/mL), A51 (105.23 IU/mL) and A60 (79.92 IU/mL) are characterized by lower titers of neutralizing antibodies. However, serum A72, which shows a high neutralizing capacity in the PRNT assay, reveals only a moderate effect in the pseudotype-based assay (121.87 IU/mL). Correlation of the PRNT and pseudotype-based assay revealed a Pearson correlation coefficient of 0.9763 (*p* = 0.0044) for the non-ARDS sera and 0.6538 (*p* = 0.1591) for the ARDS sera.

These data indicate that PRNT and pseudotype-based assays have comparable results. However, in case of the ARDS sera, differences in the titer found by PRNT are not completely resolved by the pseudotype-based assay.

Based on these data, additional analyses of the sera were performed to further characterize the immune response and to qualitatively compare the meaningfulness of the respective methods for antiserum characterization.

### 3.2. Class-Specific Quantification of RBD-Specific Antibodies by ELISA

For the ELISAs, the RBD was chosen as immobilized antigen. As the majority of neutralizing antibodies binds to the RBD, the focus on the RBD as target antigen enhances the likelihood to detect neutralizing antibodies. In case of the complete spike, a higher fraction of binding but non-neutralizing antibodies would be detected. The analysis discriminated between IgG, IgM and IgA.

**Figure 3:**
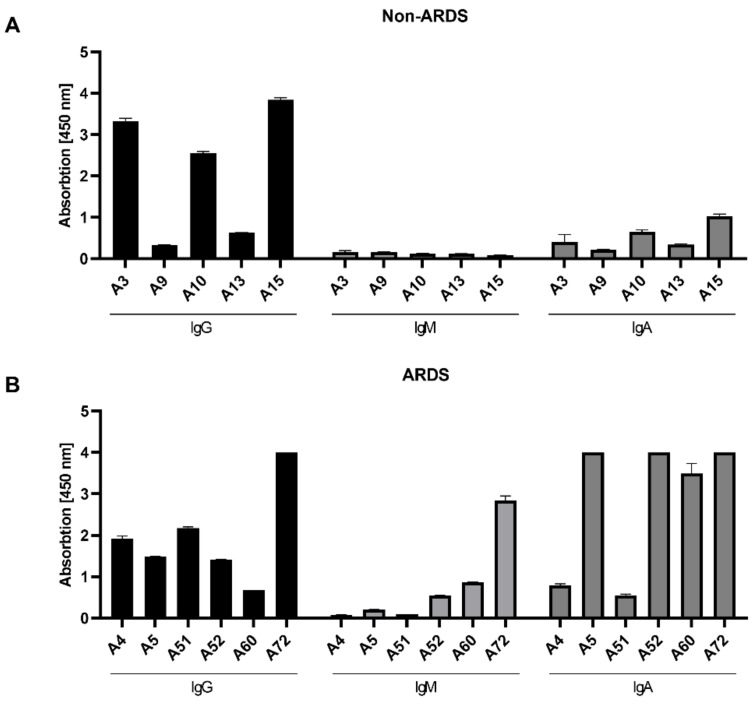
Quantification of class-specific RBD-binding antibodies by ELISA. ELISA plates were coated with the purified SARS-CoV-2 RBD (MRS), and convalescent sera from non-ARDS (**A**) and ARDS patients (**B**) were applied. RBD-specific IgG, IgM and IgA were detected. All values were measured in duplicates and are represented as mean + SD.

As observed by the neutralization assays, the analysis of the RBD-specific IgG in the sera from the non-ARDS patients (Figure 3A) revealed a heterogenous response. In 3 cases (A3 (3.33 rel. units), A10 (2.55 rel. units) and A15 (3.85 rel. units)), a high IgG titer was found; in two cases (A9 (0.33 rel. units) and A13 (0.63 rel. units)), a low titer was found. In contrast to the sera from non-ARDS patients, the IgG titer determined for the sera of ARDS patients (Figure 3B) was more homogenous (A4 (1.92 rel. units), A5 (1.48 rel. units), A51 (2.18 rel. units), A52 (1.42 rel. units)), with the exception of a lower titer in A60 (0.67 rel. units) and a higher titer in serum A72 (4 rel. units). With respect to the IgA and IgM levels, the differences between the groups were more striking (Figure 3A,B). For all analyzed sera from the non-ARDS group, the titer of RBD-specific IgM was low (below 0.2 rel. units), while in 3 sera from ARDS patients (A52, A60, A72), significant levels of IgM were detectable ranging from 0.55 to 2.84 rel. units. The RBD-binding IgA titer was, for all samples from the non-ARDS group, ≤1 rel. unit. For the sera derived from ARDS patients, sera A5 (4 rel. units), A52 (4 rel. units), A60 (3.50 rel. units) and A72 (4 rel. units) had high values above 3.5 relative units. This is in accordance with a report that described the elevated levels of spike-specific IgA in the sera of patients suffering from ARDS [24].

For the non-ARDS sera, there is a clear correlation between the titer of RBD-binding IgGs and the neutralizing activity of these sera. In case of the ARDS sera, no obvious correlation can be found between RBD-binding IgG and the neutralizing titer.

### 3.3. Analysis of Antibody Affinity to the RBD by Surface Plasmon Resonance

**Figure 4:**
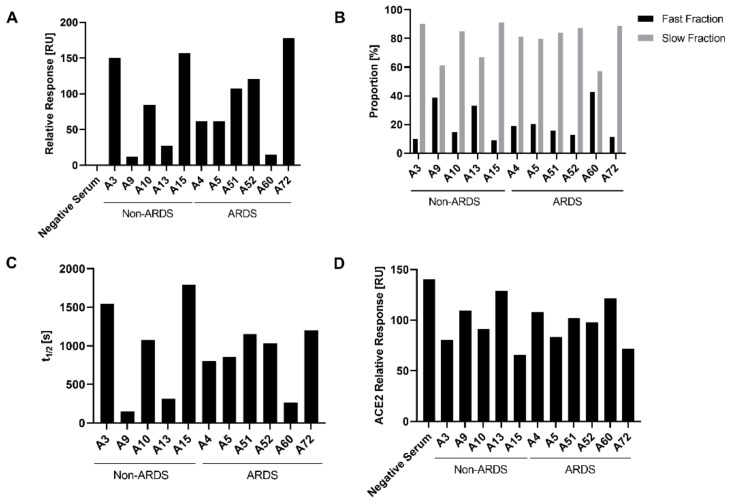
Characterization of RBD binding, stability and impact on ACE2 interaction of the convalescent sera by SPR. SARS-CoV-2 RBD (MRS) was immobilized on a CM5 sensor chip and measurements were performed using the Biacore T200 system. Sera were injected with a contact time of 180 s and 180 s dissociation. (**A**) Binding stability of the sera, defined 60 s after the end of injection. Background binding to the blank flow-cell was subtracted. The shown relative response levels are adjusted for the anti-RBD control. (**B**), Proportion of antibodies within the sera forming short-lived (fast fraction) and long-lived (slow fraction) complexes with the immobilized RBD. Calculations were performed with the Biacore Evaluation software. (**C**) Half-lives of the complexes in the respective slow fractions. (**D**) ACE2 binding stability after injection of the sera. ACE2 was applied for 60 s after the dissociation step of the sera. A duration of 60 s after end of injection, the binding stability of ACE2 was measured. Results were blank subtracted and adjusted for the anti-His control.

To further characterize the binding of antibodies to immobilized RBD, surface plasmon resonance analyses (SPR) were performed and the relative response units were determined. The relative response reflects the binding of the polyclonal antibody mixture in the sera to the immobilized RBD. Analysis of the relative response (Figure 4A) revealed, for the non-ARDS group, for the sera A9 (11.97 RU) and A13 (27.31 RU), a reduced binding as compared with sera A3 (150.15 RU), A10 (84.37 RU) and A15 (157.32 RU). The ARDS group showed a decreased binding for serum A60 (14.68 RU) as compared to sera A4 (61.87 RU), A5 (61.60 RU), A51 (107.76 RU), A52 (120.80 RU) and A72 (178.40 RU). For the non-ARDS-derived sera, the binding determined by SPR correlates with the quantification of IgGs determined by ELISA (Figure 3A). This might reflect that IgGs represent the prominent antibody fraction in the non-ARDS sera (Figure 3A).

For the ARDS sera, there is a less pronounced correlation between the IgG-specific ELISA data and the SPR-based binding data. This might be explained by the very high levels of IgA antibodies in some of the sera, that affect the observed total binding activity determined by SPR.

A relevant aspect for the neutralizing capacity of antibodies is the stability of the antigen–antibody complex. The analysis of the half-life of the antigen–antibody complexes by SPR provides a tool to determine the stability of antigen–antibody complexes. Based on this parameter, two fractions can be defined: the fast fraction, describing less stable antigen–antibody complexes, and the slow fraction, encompassing long-lived, stable complexes. The proportion of the slow fraction was thereby notably higher for the non-ARDS sera A3 and A15 and the ARDS serum A72, in contrast to the non-ARDS sera A9 and A13 and the ARDS serum A60 (Figure 4B).

Among the analyzed slow fractions, the longest half-life for the antibody–antigen complexes were observed for the non-ARDS sera A3 (t_1/2_ 1541.95 s) and A15 (t_1/2_ 1794.79 s) and for the ARDS sera A51 (t_1/2_ 1153.28 s) and A72 (t_1/2_ 1201.25 s) (Figure 4D). All these sera show a neutralization titer above 100 IU/mL. In contrast, the non-ARDS sera A9 (t_1/2_ 149.45 s) and A13 (t_1/2_ 314.65 s) and the ARDS serum A60 (t_1/2_ 260.91 s) (Figure 4D) show short half-lives in the slow fraction and are characterized by smaller neutralizing activities below 100 IU/mL. However, the ARDS sera A51 and A52, which strongly differ with respect to their neutralizing capacity, have comparable half-lives to the antibody–antigen complexes (A51: t_1/2_ 1153.28 s and A52: 1035.99 s). The same holds true for the sera A4 and A5, which have similar half-lives (A4: t_1/2_ 800.14 s and A5: t_1/2_ 683.01 s) despite differences in the neutralizing capacity. These data indicate that for the non-ARDS sera, the half-life correlates with the neutralization titer. For the ARDS sera, there is no clear correlation.

### 3.4. Characterization of the Impact of Immune Sera on the RBD-ACE-2 Interaction by Surface Plasmon Resonance

Many neutralizing antibodies interfere with the binding of the RBD to the endogenous receptor for SARS-2, ACE2. Therefore, it was investigated if the RBD-ACE2 interaction is affected by the presence of the sera. The impact of the patient sera on the binding of ACE-2 to immobilized RBD was analyzed by SPR. For the sera A3 (254.58 IU/mL) and A15 (693.67 IU/mL), which have the highest neutralizing titer of the non-ARDS sera, the strongest inhibition of the ACE2-RBD interaction can be seen. For the non-ARDS sera, there is a qualitative correlation between the data from the neutralization experiments and the impact on the ACE2-RBD interaction (A3: 80.50 RU; A15: 65.85 RU).

In case of the sera derived from the ARDS patients, there is a less clear correlation. For serum A5 (261.10 IU/mL), that has the strongest neutralizing capacity among the analyzed ARDS sera, a good correlation with the observed inhibition of the ACE2-RBD interaction (83.39 RU) can be observed. The difference in the neutralizing activity between serum A51 (105.23 IU/mL) and serum A52 (242.15 IU/mL) is very weakly reflected by the data describing the impact on the ACE2-RBD interaction (A51: 102.13 RU and A52: 97.77 RU). For serum A72, which has a moderate neutralizing activity in the pseudotype-based assay (121.87 IU/mL) but a stronger neutralizing effect in the PRNT assay (PRNT_50_ = 640), a strong inhibition of the ACE2-RBD interaction (71.60 RU) was found. This could be due to the high titer of RBD-specific IgG and IgA.

Taken together, these data indicate a correlation between the IgG titer of RBD-specific antibodies, the stability of the antigen–antibody complexes, the inhibition of the ACE2-RBD interaction and the neutralizing capacity for the non-ARDS sera. For the ARDS sera, this correlation is less pronounced.

### 3.5. Inhibition of Syncytium Formation as a Tool to Characterize Spike-Specific Neutralizing Antisera

The PRNT-based neutralization assay is the gold standard for the analysis of the neutralizing activity of sera and antibodies [11]. However, in case of SARS-CoV-2, these experiments require access to a BSL3 facility.

In light of this, it was investigated if the syncytium formation assay (SFA) could be a simple and robust surrogate method to be performed instead of classic virus neutralization assays. On the one hand, the SFA depends on intact ACE2 and, on the other hand, it depends on the spike protein as a ligand that is properly inserted into the cellular plasma membrane, ensuring correct folding. In contrast to the analysis of the impact of antibodies on the ACE2-RBD interaction, this assay should allow an analysis of further steps of the viral entry process, especially the fusion process. If there is an ACE2–spike interaction in this experimental system, the spike-immanent fusogenic activity causes fusion of the ACE2-expressing cells with the spike-expressing cells. This process mimics several steps of the viral entry process and depends on functional spike and ACE2.

**Figure 5:**
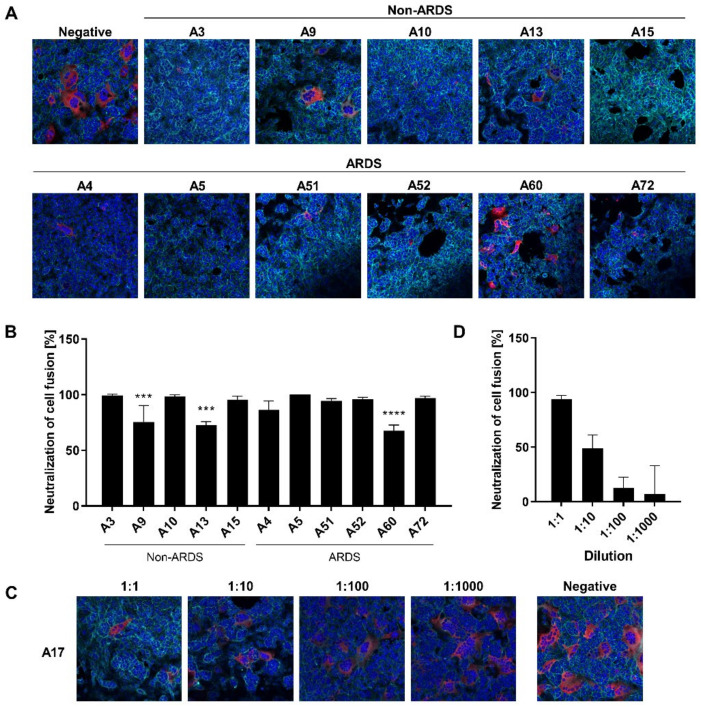
Analysis of the neutralizing capacity of patient sera on spike-dependent syncytia formation. SARS-CoV-2-Spike transfected HEK293T cells and endogenously ACE2-expressing Vero cells were co-cultured in presence of convalescent or negative control sera and analyzed for syncytium formation. (**A**) Confocal laser scanning microscopy (CLSM) images 24 h after start of the co-culture. Spike protein was visualized using a specific antibody (red), the actin cytoskeleton was stained with Phalloidin-Atto 633 (cyan) and nuclei were stained with DAPI (blue). (**B**) Neutralization of cell fusion was calculated as reduction in the proportion of nuclei found within spike-positive syncytia compared with the syncytia formation after negative serum incubation. Calculations are based on quantification of syncytia in three fields of view. Data are shown as mean + SD. Significance of the results was analyzed by two-way ANOVA with respect to the serum with the highest neutralization of cell fusion (A5). *** *p* ≤ 0.001; **** *p* ≤ 0.0001. (**C**) Co-culture was carried out in the presence of serial dilutions of convalescent serum A17 (1:1–1:1000) and CLSM analyses were performed as described in (**A**). The corresponding control in presence of negative serum is shown. (**D**) Neutralization of cell fusion of the serial dilutions of convalescent serum A17 depicted in (**C**). Calculations were performed as described in (**B**).

For the sera derived from non-ARDS patients, there is a clear qualitative correlation between the results of the SFA and the “classic” neutralization assays. The sera A9 (68.09 IU/mL) and A13 (99.10 IU/mL), which display a lower neutralization capacity in the neutralization assays as compared with the other sera, show a reduced neutralization of the cell fusion in the SFA (Figure 5A,B). This is also the case for low titer ARDS sera as observed for A60 (79.2 IU/mL), which has a decreased neutralization activity in the SFA (Figure 5A,B). To investigate whether the SFA is suitable to resolve quantitative correlation between antibody titer and neutralization of cell fusion, serum A17 was serially diluted and analyzed by SFA (Figure 5C,D). The assay reveals, for three dilution steps, a linear correlation between concentration and inhibition of syncytium formation.

Taken together, these data indicate that the SFA-based data are qualitatively in line with the data from the neutralization assays.

## 4. Discussion

This study aimed to compare different methods for analysis of the humoral immune response. In light of a rapidly increasing number of vaccinated and convalescent individuals, it is of major relevance to develop robust methods to evaluate the neutralizing antibody titer as a prognostic marker. Although, so far, there is no correlate of protection defined [10], there is a need for robust methods to determine the titer of neutralizing antibodies. ELISA-based approaches allow the convenient determination of antibody titers, but binding antibodies are not automatically identical with neutralizing antibodies. In principle, this can be improved by selection of antigen domains that are preferentially recognized by neutralizing antibodies, but this approach is limited by structural considerations: many epitopes are not based on sequential motives, but on a defined 3D structure. Nevertheless, determination of the titer and stratification of Ig classes is an important parameter for the analysis of the immune response. In this study, an obvious difference between sera derived from ARDS patients and non-ARDS patients was observed. Sera from ARDS patients are more complex and contain higher amount of RBD-binding IgM and especially IgA. This is in accordance with previous reports [25,26,27]. Especially in the cases of some of the sera derived from ARDS patients (sera A5 and A52), a high titer of spike-specific IgAs could be detected while the IgG titer was low. As these sera display a high neutralizing capacity, it was concluded that the spike-specific IgA significantly contributes to the neutralizing activity of these sera.

Biophysical assays, such as surface plasmon resonance, represent a helpful tool to significantly deepen the analysis of the immune response [28]. In addition to a detailed quantitative analysis of antibody–antigen interactions, additional parameters, such as the stability of these complexes, can be observed. As a variety of neutralizing antibodies interferes with the spike–ACE2 interaction [29], the detailed analysis of the impact of antisera on this interaction is relevant for the characterization of the neutralizing capacity.

The biophysical analysis of the spike-specific immune response in principle describes protein–protein interactions and their modulation. In contrast to virus neutralization assays using infectious viral particles or pseudotyped vectors, biophysical assays allow a better standardization as compared with cell-culture-based assays. However, virus neutralization by antibodies depends on a variety of parameters: here it should be considered that apart from the interference with the RBD-ACE2 interaction, additional processes can be affected by neutralizing antibodies, i.e., the activation and functionality of the fusogenic sequence [30,31]. Assays that are exclusively focused on the ACE2-RBD interaction fail to address this point. The SFA [15] could represent an interesting tool to broaden the analysis of neutralizing antibodies, as it depends on intact spike and ACE2. In contrast to the in vitro assays based on purified proteins, it can be assumed that ACE2 and the overexpressed spike are properly folded in the cells used for the SFA.

Comparing the results of the classic neutralization test to the ELISA and SPR data, for the non-ARDS data, there is an obvious correlation. This indicates that these methods could represent an additional tool for a detailed and well-standardized analysis of immune sera without the requirement of BSL3 facilities. On the other hand, for the sera of the ARDS patients, there is a less clear correlation between ELISA and SPR data and between ELISA and classic neutralization data. The higher proportion of IgM and especially of RBD-binding IgA and neutralizing antibodies that do not bind to the RBD might be causative factors, which make the analysis by these additional parameters much more complex and thereby limit the general application of SPR-based methods as a general surrogate system for analysis of neutralizing antibodies.

On the one hand, a major aspect of this study is the broad variety of different methods that was comparatively used to test these sera. This sets our study apart from previous studies [32,33,34], which focused on fewer assays. On the other hand, the complexity of the methodological approach limits the number of samples that could be analyzed. It should be clarified that the aim of the study is not the detailed quantification of large numbers of sera from different donors but the characterization of the suitability of methods for sera of different complexity. For this purpose, complex sera with a higher fraction of IgM and especially IgA-specific antibodies, which were found among the ARDS-derived sera, were compared to sera containing very high fractions of IgGs. We could observe that complex sera, which are characterized by higher levels of IgM, and especially IgA, require the use of classic neutralization assays for characterization of their neutralizing capacity. Although the number of analyzed sera was limited, the sera A5 and A52, which are characterized by higher levels of RBD-specific IgA, show no clear correlation for the data of the neutralization assays with the data from the SPR-based assays. This indicates that IgA-specific antibodies are a relevant factor for the neutralizing capacity, but the chosen design of the SPR-based approach was not suitable to reflect this.

In order to characterize the immune response of vaccinated and convalescent individuals, there is an urgent need for robust and highly reproducible methods for determination of the neutralizing capacity of sera and antibodies. This study describes that, for preselected sera (non-ARDS patients), a combination of SPR-based approaches or SFA-based assays could be used as an alternative to virus-based neutralization assays, but further work is required to broaden the application to more complex sera.

## Figures and Tables

**Table 1:** Overview of sera obtained patients suffering from COVID-19 and their clinical outcome.

Sample	Age	Sex	Group	Symptoms to Hospit.	Days to ICU	ICU Days	Fever Days	Vent Days	Oxygen Days	ECMO Days	Hosp Days
A3	47	M	non ARDS	7	n/a	n/a	10	n/a	25	n/a	28
A4	69	W	ARDS	0	3	19	11	14	31	n/a	39
A5	58	M	ARDS	5	7	37	40	27	33	n/a	42
A9	83	M	non ARDS	2	n/a	n/a	0	n/a	15	n/a	28
A10	82	M	non ARDS	8	n/a	n/a	8	n/a	29	n/a	31
A13	80	M	non ARDS	6	n/a	n/a	7	n/a	7	n/a	18
A15	77	M	non ARDS	4	n/a	n/a	4	n/a	11	n/a	12
A51	67	M	ARDS	0	2	90	32	90	92	23	92
A52	48	M	ARDS	5	5	87	10	84	87	81	87
A60	77	W	ARDS	3	3	34	13	26	34	n/a	56
A72	27	M	ARDS	n/a	n/a	n/a	n/a	n/a	n/a	n/a	44

## Data Availability

All supporting data are included in this manuscript.
